# Molecular Identification of Species Belonging to *Culex vishnui* Subgroup (Diptera: Culicidae), Vectors of Japanese Encephalitis Virus, in Taiwan

**DOI:** 10.4269/ajtmh.23-0285

**Published:** 2024-09-10

**Authors:** Han-Hsuan Chung, Tien-Huang Chen, Pei-Feng Wang, Yoshio Tsuda, Hwa-Jen Teng, Shiu-Ling Chen

**Affiliations:** ^1^ Center for Diagnostic and Vaccine Development, Centers for Disease Control, Ministry of Health and Welfare, Taipei, Taiwan;; ^2^ Department of Medical Entomology, National Institute of Infectious Diseases, Tokyo, Japan

## Abstract

Classification of mosquitoes with overlapping features remains problematic when using traditional morphological identification alone. In this study, we used molecular methods to elucidate the taxonomic status of *Culex tritaeniorhynchus*, *Culex annulus*, and *Culex pseudovishnui* species as vectors of the Japanese encephalitis virus belonging to the *Culex vishnui* subgroup and gene flow among them. In this study, 76, 59, and 3 samples of *Cx. annulus*, *Cx. tritaeniorhynchus*, and *Cx. pseudovishnui*, respectively, were collected around Taiwan. Phylogenetic analysis and genetic divergence were based on genomic sequence variations in ribosomal DNA and the internal transcribed spacer (rDNA) and cytochrome c oxidase subunit I (COI). Our results revealed that *Cx. annulus* and *Cx. vishnui* are genetically similar and share a gene pool among the species from Taiwan and other Asian countries. However, two hidden taxa of *Cx. tritaeniorhynchus*, which clustered together according to the rDNA sequences, were discovered based on the COI sequences. In addition, *Cx. pseudovishnui* has different gene pools from those of the strains from other countries, implying that the population from Taiwan is probably either a unique strain or a sibling species. This study provides molecular information on the taxonomic status of the species in the *Cx. vishnui* subgroup in Taiwan and gene flow between these species, providing valuable information for vector control operations and the delineation of the evolutionary process.

## INTRODUCTION

Japanese encephalitis is a mosquito-borne disease caused by the Japanese encephalitis virus (JEV) with high mortality and irreversible neurological damage. Taiwan has 132 mosquito species belonging to 17 genera of the family Culicidae,^1^ of which *Culex tritaeniorhynchus* Giles, *Culex annulus* Theobald, and *Culex fuscocephala* Theobald are local vectors of JEV.^2^ These two former species, along with *Culex pseudovishnui* Colless, are members of the *Culex vishnui* subgroup of the *Culex sitiens* group. In addition, this subgroup includes *Culex perplexus* Leicester, *Culex alienus* Colless, *Culex philippinensis* Sirivanakarn, *Culex whitei* Barraud, and *Culex incognitus* Baisas distributed in other countries.^3^ However, these species have similar morphologies and breeding sites, which were easily confused for species identification.

For example, *Cx. annulus* was first recognized in 1960 and identified as the dominant species of the JEV vector in Taiwan; however, it is easily confused with *Culex vishnui* Theobald and *Cx*. *tritaeniorhynchus* based on morphological features.^4^^–^^6^ Later, *Cx. tritaeniorhynchus* replaced *Cx. annulus*, emerging as the dominant population in the field and playing a crucial role in JEV transmission over nearly two decades.^2^^,^^7^^–^^9^ Potential for the transovarial transmission of JEV in *Cx. tritaeniorhynchus* has strengthened its importance in spreading JEV.^10^^,^^11^ Although previous reports have described the detection of JEV in *Cx. pseudovishnui* in India and Sri Lanka,^12^^,^^13^ this species in Taiwan has a relatively low density and is not believed to be a JEV vector. Because the role of different species with different vector competencies in JEV transmission is crucial for risk assessment, accurate species classification of these morphologically similar mosquitoes is necessary for vector control operations and disease prevention.

Mosquitoes and pathogens can enter the world via transportation or human migration. For example, *Aedes albopictus* Skuse invaded America through the extensive trade of waste tires and shipments of fortune-inviting bamboo (*Dracaena* spp.).^14^ Airport malaria in Belgium has raised the possibility of introducing disease-carrying vectors via transportation.^15^ The introduction of exotic mosquito species into international airports has been monitored in The Netherlands.^16^ Furthermore, the introduction of the West Nile virus into New York City from the Eastern Hemisphere has been documented.^17^ On the other hand, five genotypes of JEV have been recognized, and each of them has its own geographic distribution.^18^ However, the major role of JEV genotype III in Taiwan was replaced by genotype I, which is prevalent in Japan and China, and spread throughout the island.^9^^,^^19^ Therefore, identifying vector mosquitoes to track vector origin or interaction with pathogens is necessary, whether based on morphological characteristics or other methods.

Owing to advances in molecular biotechnology and the availability of genome sequencing, molecular identification has been developed for decades to accurately identify species, especially insects with morphologically indistinguishable features.^20^ Diagnostic polymerase chain reaction (PCR) based on genomic sequence variations was established for species identification. For example, the ribosomal DNA and the internal transcribed spacer (rDNA) have been used to separate the morphologically similar mosquito species *Anopheles freeborni* and *Anopheles hermsi* and *Aedes aegypti* and *Aedes albopictus*.^21^^,^^22^ A similar method was used to differentiate the morphologically similar *Culex pipiens* complex.^23^ In addition, species-specific PCR based on the internal transcribed spacer sequence was used to distinguish *Cx. vishnui*, *Cx*. *tritaeniorhynchus*, and *Cx*. *pseudovishnui* belonging to the *Cx. vishnui* subgroup.^22^^,^^24^ In addition to rDNA, cytochrome c oxidase subunit I (COI) from the mitochondrial genome, which is complementary to rDNA, was co-assessed in species molecular identification.^25^ Studies have successfully distinguished the *Cx. vishnui*, *Cx*. *tritaeniorhynchus*, and *Cx*. *pseudovishnui* and other species in various regions by COI sequence.^26^^–^^29^ Recently, Arai et al.^30^ analyzed the COI sequence and separated the *Cx*. *tritaeniorhynchus* into two genetically independent taxa, suggesting this marker exhibits higher resolution for species identification.

In this study, we aimed to establish a molecular method for identifying species belonging to the *Cx. vishnui* subgroup in Taiwan, including *Cx. annulus, Cx. tritaeniorhynchus*, and *Cx. pseudovishnui*, and elucidate gene flow in these species to evaluate the chances of success of invading vectors.

## MATERIALS AND METHODS

### Mosquito samples.

Mosquitoes were collected from various locations in Taiwan using light traps between 2011 and 2012. Mosquito species were identified based on established morphological features under a dissecting microscope by experienced medical entomologists.^1^^,^^31^ The samples belonging to *Cx*. *annulus*, *Cx*. *tritaeniorhynchus*, and *Cx*. *pseudovishnui* species were used for further experiments. In addition, a total of 19 samples of *Cx*. *tritaeniorhynchus* (15), *Cx*. *vishnui* (three), and *Cx*. *pseudovishnui* (one) species samples were obtained from the National Institute of Infectious Diseases of Japan. These samples were stored in a −80°C freezer until DNA extraction.

### Mosquito DNA extraction.

The detailed protocol has been described previously.^24^ Each mosquito was placed in a 1.5-mL tube with 180 *µ*L Dulbecco’s phosphate-buffered saline (Gibco, Thermo Fisher Scientific, Waltham, MA) and one glass bead (diameter of 3 mm) (Hycell International Co. Ltd., Taipei, Taiwan). The samples were homogenized three times using a TissueLyser (Qiagen, Hilden, Germany) at a frequency of 30 strokes/s with shaking for 30 seconds. The homogenized samples were processed using the QIAamp Tissue Kit (Qiagen) following the manufacturer’s guidelines. The genomic DNA was eluted in 40 *µ*L Tris-Ethylenediaminetetraacetic acid buffer for immediate use and stored in a −20°C refrigerator.

### Molecular sequencing.

Phylogenetic analyses were performed based on rDNA genes described in a previous study.^24^ A PCR product flanked by partial sequences of 18S rRNA and internal transcribed spacer 2 (ITS2) and complete sequences of 5.8S rRNA and ITS1, was amplified using 18SF (5′-GTAAGCTTCCTTTGTACACACCGCCCG-3′) and 28SR1 (5′-GGGGTAGTCACACATTATTTG-3′) primer sets. The amplification of rDNA was conducted by using Taq DNA Polymerase (Cat. No. 10342053; Invitrogen, Carlsbad, CA). The program consisted of one cycle at 95°C for 3 minutes, 40 cycles of denaturation at 95°C for 30 seconds, annealing at 52°C for 30 seconds, extension at 72°C for 1 minute, and final extension at 72°C for 4 minutes. Amplicons were cloned into *p*ZBack/blunt vector using a ZBack Faster Ligation Kit (BIOTOOLS Co., Ltd., New Taipei, Taiwan) following the manufacturer’s recommendations. The other marker, COI, was analyzed according to a previous study.^32^ A PCR product was amplified using LCO1490 (5′-GGTCAACAAATCATAAAGATATTGG-3′) and HCO2198 (5′-TAAACTTCAGGGTGACCAAAAAATCA-3′) primer sets. The amplification of COI was conducted by SapphireAmp Fast PCR Master Mix (Cat. No. RR350; Takara Bio Inc., Shiga, Japan). The program consisted of one cycle at 94°C for 1 minute, 44 cycles of denaturation at 98°C for 5 seconds, annealing at 55°C for 8 seconds, extension at 72°C for 5 seconds, and final extension at 72°C for 10 minutes. The selected clone of rDNA and amplicons of COI were sent out for Sanger sequencing (Genomics, New Taipei, Taiwan) with kit-specific primers and 18SF, 28SR1, 18SF4 (5′-GGCTGGTCAGTCTATATCGC-3′), and 5.8SR1 (5′-TTGCGGATGACCAGTCG-3′) for rDNA or LCO1490 for COI. Moreover, sequences from National Center for Biotechnology Information (NCBI) GenBank included in this study are listed in Supplemental Material 1.

### Phylogenetic analysis and sequence divergence.

Phylogenetic analysis and intra- and interspecific divergence were performed using MEGA11: Molecular Evolutionary Genetics Analysis v. 11.^33^ Briefly, rDNA and COI sequences were trimmed for alignment using MUSCLE and the neighbor-joining (NJ) and maximum likelihood (ML) methods based on the model with the lowest Bayesian information criterion.^25^^,^^34^^,^^35^ The pairwise deletion was used to treat missing data. The bootstrap values were estimated using 1,000 replicates. One representative specimen was selected for analysis when multiple sequence specimens shared the same haplotype. The BLASTn suite for multiple sequence BLAST in GenBank (https://blast.ncbi.nlm.nih.gov/Blast.cgi) was used to calculate the identity of sequences between different specimens to ascertain sequence divergence.

## RESULTS

**Figure 1.**
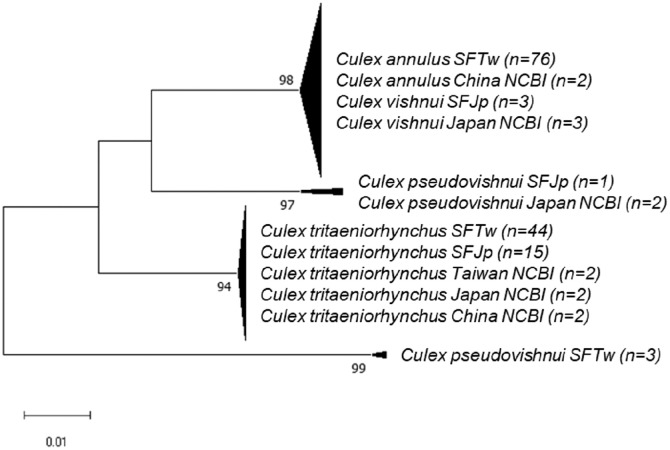
Phylogenetic analysis of *Culex vishnui* subgroup based on ribosomal DNA and the internal transcribed spacer (rDNA) sequences, using neighbor-joining. Pairwise deletion, leading to 708 positions of sequences in the final dataset, were analyzed. The rate variation among sites was modeled with a γ distribution (shape parameter 0.14). The bootstrap value (1,000 replicates) above 70 is shown at nodes. Taxa within the same monophyletic clade were compressed. Samples from Taiwan and Japan are abbreviated as SFTw and SFJp, respectively. NCBI = National Center for Biotechnology Information.

The sequences obtained in this study and those retrieved from the NCBI database were used to construct the phylogenetic tree of the *Cx. vishnui* subgroup and are listed in Table 1 and Supplemental Table 1. Representative sequences were deposited in NCBI GenBank (accession numbers: OQ674278-OQ674343 and PP572885-PP572901). The NJ phylogenetic tree recovered from rDNA sequences was divided into four strongly supported monophyletic clades of *Cx. vishnui*/*Cx. annulus*, *Cx*. *tritaeniorhynchus,* and *Cx*. *pseudovishnui* from Taiwan and Japan (bootstrap value >94) (Figure 1). Notably, *Cx*. *annulus* strains from Taiwan and China and *Cx. vishnui* from Japan were placed in the same clade, with a bootstrap value of 98, suggesting that *Cx*. *annulus* and *Cx. vishnui* strains shared the same gene pool of rDNA genes. On the other hand, *Cx*. *tritaeniorhynchus* strains from Taiwan, Japan, and China also grouped together. To our surprise, *Cx*. *pseudovishnui* mosquitoes from Taiwan and Japan were divided into two distinct clades, suggesting that these two species evolved into different lineages. When we constructed the tree based on COI, similar results were observed. *Culex annulus* from Taiwan grouped with *Cx. vishnui* from other countries. *Culex pseudovishnui* from Taiwan and Japan were divided into different clades. To our surprise, *Cx. tritaeniorhynchus* was separated into two independent clades with a bootstrap value of 99.

**Table 1:** Origin and number of *Culex vishnui* subgroup analyzed in this study

Species	Sampling Site	Number	Sample Origin
*Culex annulus*	Taipei (TPE)	9	Light Trap
	Yilan (YIL)	4	Light Trap
	Nantou (NAN)	22	Light Trap
	Yunlin (YUN)	5	Light Trap
	Changhua (CHC)	3	Light Trap
	Tainan (TAN)	3	Light Trap
	Kaohsiung (KAO)	6	Light Trap
	Pingtung (PTH)	1	Light Trap
	Hualien (HUA)	4	Light Trap
	Taitung (TAI)	19	Light Trap
	Total	76	–
*Culex tritaeniorhynchus*	Taipei (TPE)	4	Light Trap
	New Taipei (NTP)	3	Light Trap
	Yilan (YIL)	1	Light Trap
	Taichung (TAC)	5	Light Trap
	Changhua (CHC)	6	Light Trap
	Tainan (TAN)	6	Light Trap
	Kaohsiung (KAO)	5	Light Trap
	Pingtung (PTH)	6	Light Trap
	Hualien (HUA)	8	Light Trap
	Japan	15	NIID
	Total	59	–
*Culex pseudovishnui*	Nantou (NAN)	3	Light Trap
	Japan	1	NIID
	Total	4	–
*Culex vishnui*	Japan	3	NIID

NIID = National Institute of Infectious Diseases, Japan

**Figure 2.**
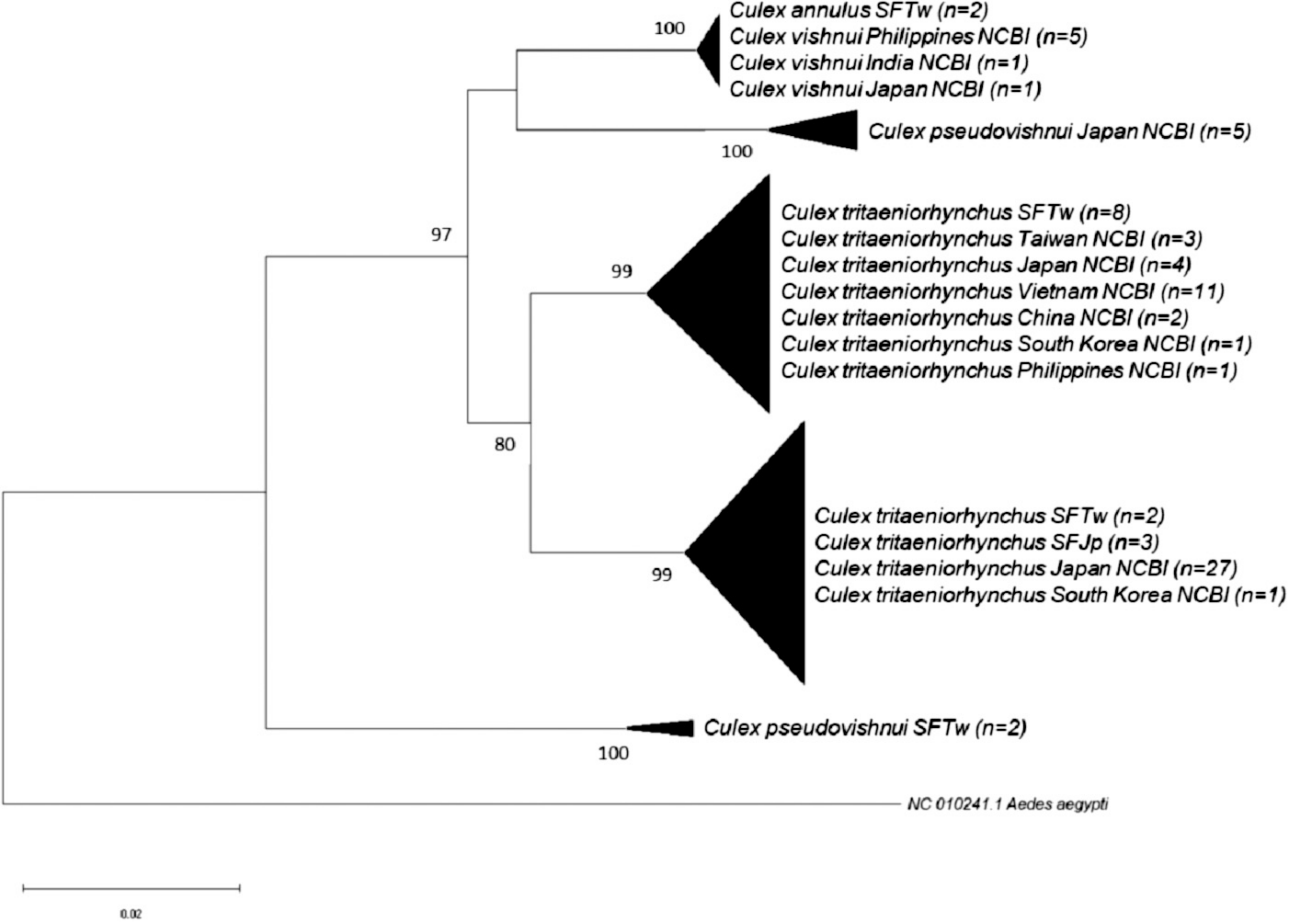
Phylogenetic analysis of the *Culex vishnui* subgroup based on cytochrome c oxidase subunit I (COI) sequences, using neighbor-joining. Pairwise deletion, leading to 614 positions of sequences in the final dataset, were analyzed. The rate variation among sites was modeled with a γ distribution (shape parameter 0.19). The bootstrap value (1,000 replicates) above 70 is shown at nodes. Taxa within the same monophyletic clade were compressed. Samples from Taiwan and Japan are abbreviated as SFTw and SFJp, respectively. Sequence of *Aedes aegypti* was included as an outgroup. NCBI = National Center for Biotechnology Information.

**Figure 3.**
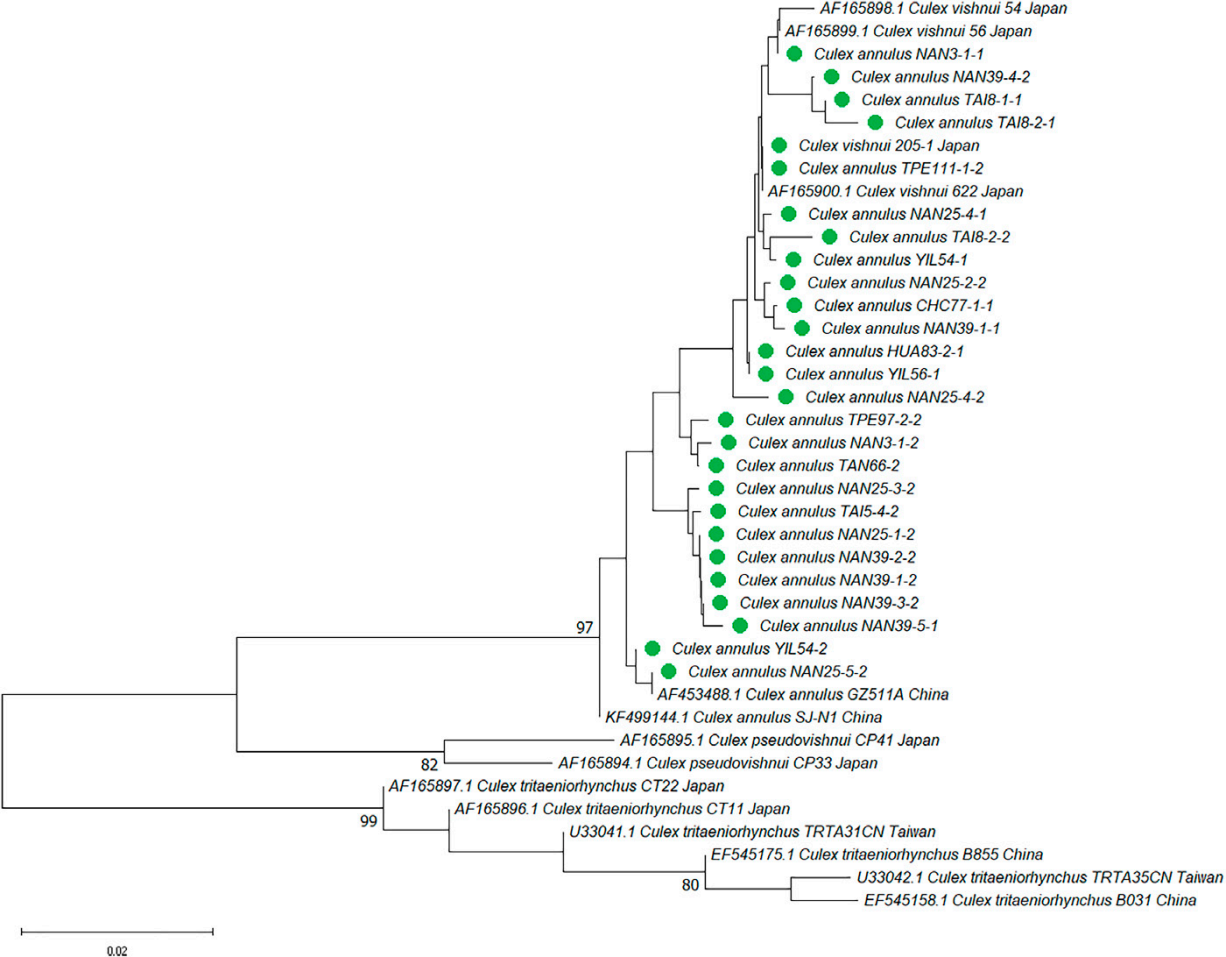
Phylogenetic analysis of *Culex vishnui and Culex annulus* based on ribosomal DNA and the internal transcribed spacer (rDNA) sequences using neighbor-joining. Pairwise deletion, leading to 679 positions of 40 nucleotide sequences in the final dataset, were analyzed. The rate variation among sites was modeled with a γ distribution (shape parameter 0.05). The bootstrap value (1,000 replicates) higher than 70 is shown at nodes. Green circles indicate the samples collected in this study. In annotation after species name, for example, TPE-A-B-C, TPE indicates the city of collection site, A indicates the random number for each collection site, B indicates the ordinal number of mosquitoes in each collection site, C indicates the two haplotypes of individual mosquito.

**Figure 4.**
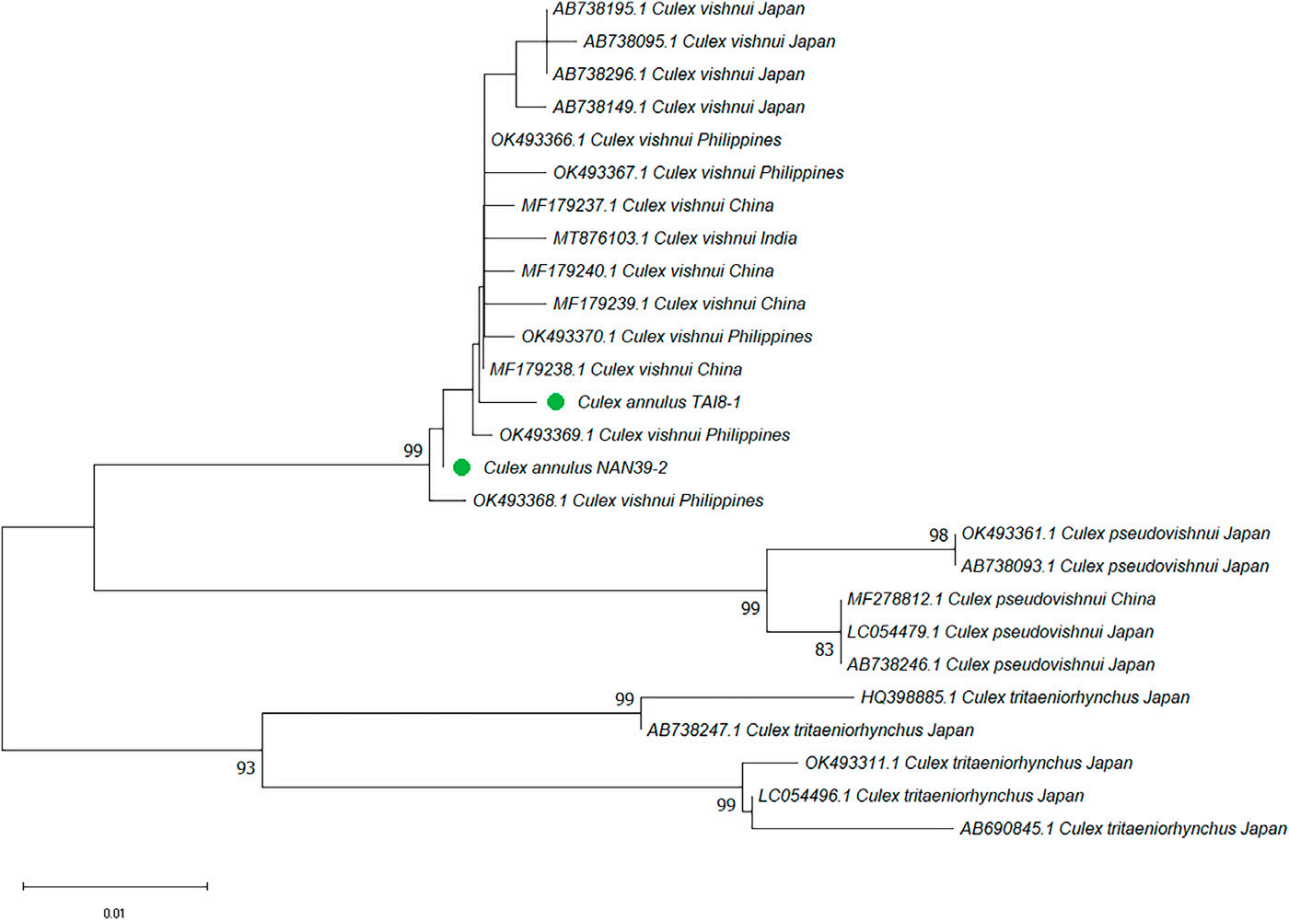
Phylogenetic analysis of *Culex vishnui *and* Culex annulus* based on cytochrome c oxidase subunit I (COI) sequences using neighbor-joining. Pairwise deletion, leading to 618 positions of 26 nucleotide sequences in the final dataset, were analyzed. The rate variation among sites was modeled with a γ distribution (shape parameter 0.16). The bootstrap value (1,000 replicates) higher than 70 is shown at nodes. The green circle indicates the samples collected in this study. In annotation after species name, for example, TPE-A-B, TPE indicates the city of the collection site, A indicates the random number for each collection site, and B indicates the ordinal number of mosquitoes in each collection site.

To further analyze the strains grouped in Figures 1 and 2, the NJ and ML trees were constructed. Generally, trees based on both methods presented similar tree topology, and the NJ trees are shown in the main text. The phylogenetic trees based on rDNA were constructed using representative haplotypes of *Cx*. *annulus* and *Cx. vishnui* strains across various countries. All of the *Cx*. *annulus* and *Cx. vishnui* grouped into one clade (bootstrap value >97). Although some sequences grouped with *Cx*. *annulus* strains from China and the others grouped with *Cx. vishnui* strains from Japan, no significant differences were observed (Figure 3; Supplemental Figure 1). The rDNA sequences of the *Cx*. *annulus* strains from Taiwan shared >97% identity with the sequences retrieved from the NCBI database of the *Cx. vishnui* strain from Japan (CV-622) and the *Cx*. *annulus* strain from China (CA-GZ511A) (Table 2). The sequences of TPE111-1-2, NAN39-3-1, and TAI8-4-2 were identical to those of CV-622. The sequence of NAN25-5-2 was identical to that of CA-GZ511A. When we constructed the trees based on COI, similar results were observed. The representative sample of *Cx. annulus* from Taiwan formed an independent clade (bootstrap value >99) with *Cx. vishnui* from Japan, Philippines, and India (Figure 4 and Supplemental Figure 2). The COI sequences of the *Cx*. *annulus* strains from Taiwan shared >99% identity with the sequences retrieved from the NCBI database of the *Cx. vishnui* strain from Japan and Philippines and the *Cx*. *annulus* strain from China. This result strongly indicates frequent genetic interflow and high sequence similarity between *Cx*. *annulus* strains from Taiwan and China and the *Cx. vishnui* strain from Asian countries.

**Table 2:** Comparison of rDNA sequence of *Culex annulus* from Taiwan with those in China *Culex annulus* and Japan *Culex vishnui*

Identity (%)	CV-622	Accumulative Number	Accumulative Percentage (%)	CA-GZ511A	Accumulative Number	Accumulative Percentage (%)
100	3	1	3.9	1	1	1.3
99	54	57	75	14	15	19.7
98	6	63	82.9	46	61	80.3
97	13	76	100	15	76	100

CA-GZ511A = China *Culex annulus*; CV-622 = Japan *Culex vishnui*; rDNA = ribosomal DNA and the internal transcribed spacer.

**Figure 5.**
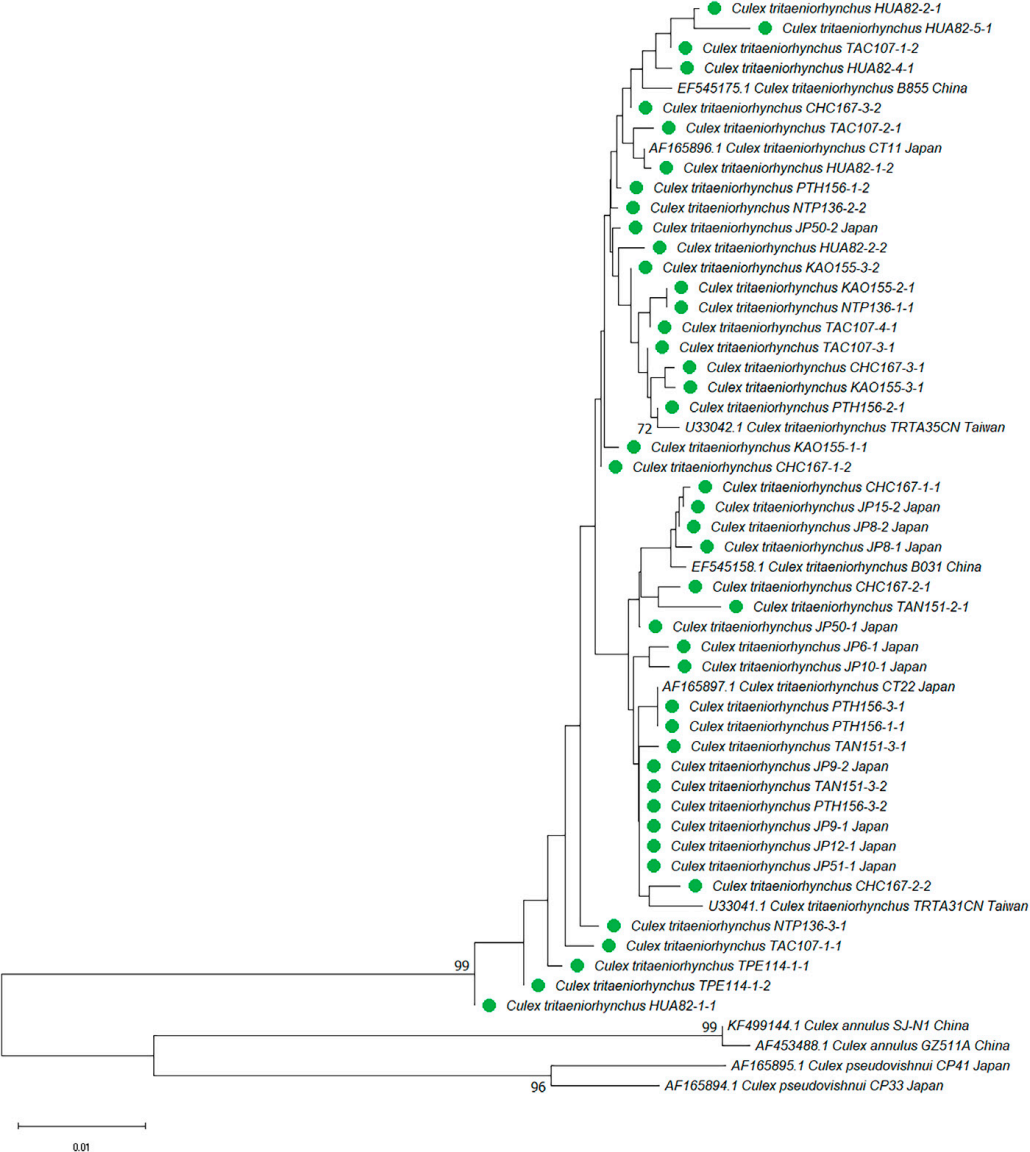
Phylogenetic analysis of *Culex tritaeniorhynchus* based on ribosomal DNA and the internal transcribed spacer (rDNA) sequences using neighbor-joining. Pairwise deletion, leading to 696 positions of 55 nucleotide sequences in the final dataset, were analyzed. The rate variation among sites was modeled with a γ distribution (shape parameter 0.07). The bootstrap value (1,000 replicates) above 70 is shown at nodes. The green circle indicates the samples collected in this study. In the note followed by species name, for example, TPE-A-B-C, TPE indicates the city of collection site, A indicates the random number for each collection site, B indicates the ordinal number of mosquitoes in each collection site, and C indicates the two haplotypes of individual mosquito.

**Figure 6.**
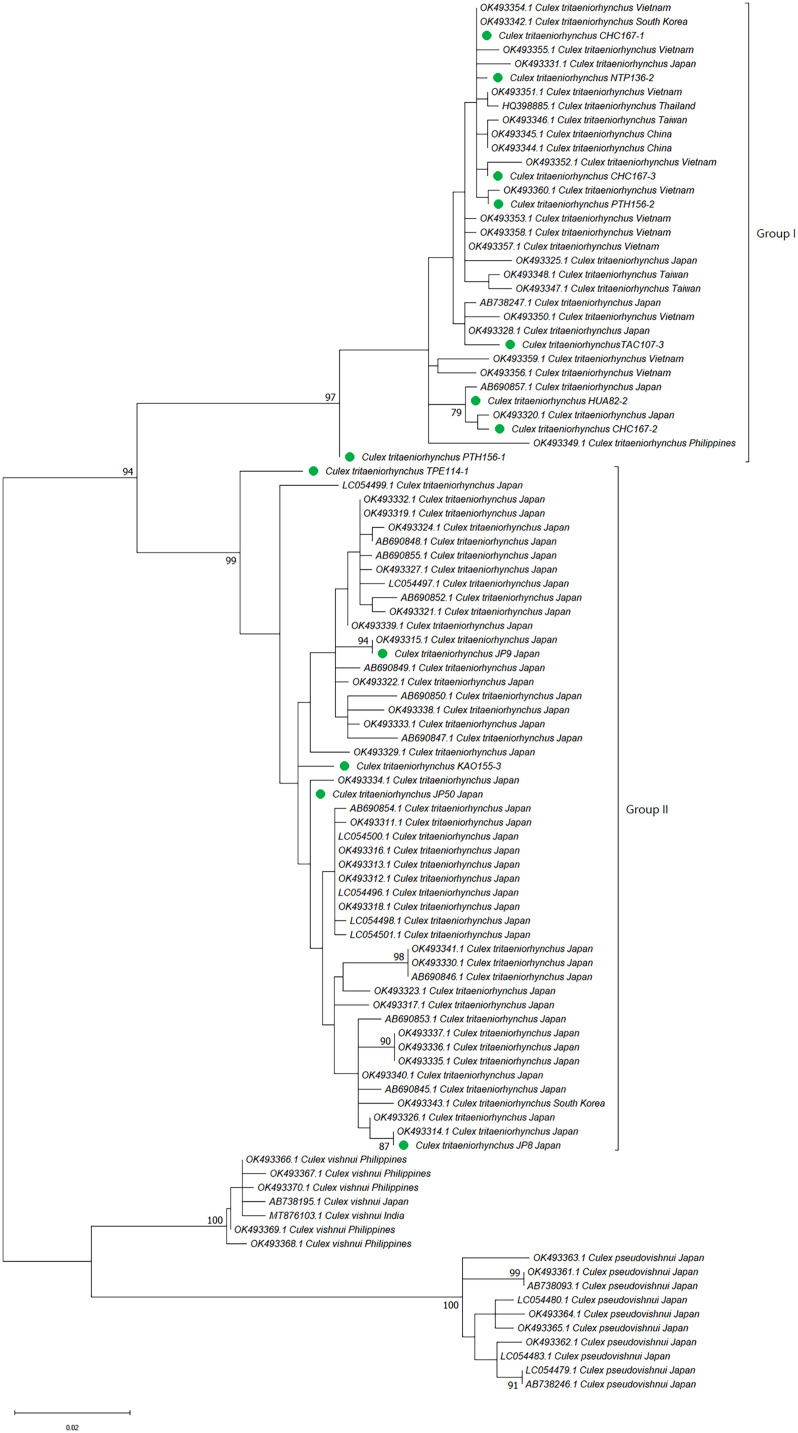
Phylogenetic analysis of *Culex tritaeniorhynchus* based on cytochrome c oxidase subunit I (COI) sequences using neighbor-joining. Pairwise deletion, leading to 587 positions of 99 nucleotide sequences in the final dataset, were analyzed. The rate variation among sites was modeled with a γ distribution (shape parameter 0.14). The bootstrap value (1,000 replicates) above 70 is shown at nodes. The green circle indicates the samples collected in this study. In the note followed by species name, for example, TPE-A-B, TPE indicates the city of collection site, A indicates the random number for each collection site, B indicates the ordinal number of mosquitoes in each collection site.

The phylogenetic trees were constructed using representative rDNA haplotypes of *Cx*. *tritaeniorhynchus* strains from Taiwan, Japan, and China (Figure 5 and Supplemental Figure 3). The sequences from different countries showed a significant overlap and mixing of sequences. The samples from the same country failed to form distinct clades. The result suggested that there is no clear geographical boundary between the samples. However, when we constructed the trees based on COI, two genetically different clades were observed (Figure 6 and Supplemental Figure 4). Some sequences for *Cx. tritaeniorhynchus* strains from Taiwan were grouped with the sequences of *Cx. tritaeniorhynchus* from Japan (Group II, bootstrap value >99), and others were grouped with *Cx. tritaeniorhynchus* from other Asian countries such as Vietnam, Thailand, Philippines, and China (Group I, bootstrap value >97). These results suggested the hidden taxa under morphologically determined *Cx. tritaeniorhynchus* were revealed in Taiwan according to COI sequence and intimated a genetic interflow between the *Cx*. *tritaeniorhynchus* strains from Taiwan and other countries.

**Figure 7.**
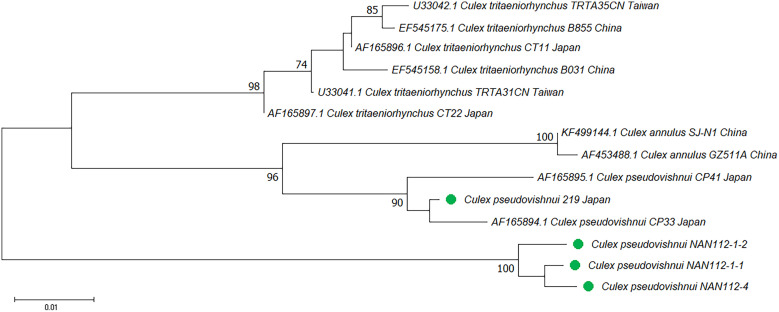
Phylogenetic analysis of *Culex pseudovishnui* based on ribosomal DNA and the internal transcribed spacer (rDNA) sequences using neighbor-joining. Pairwise deletion, leading to 692 positions of 14 nucleotide sequences in the final dataset, were analyzed. The rate variation among sites was modeled with a γ distribution (shape parameter 0.15). The bootstrap value (1,000 replicates) above 70 is shown at nodes. The green circle indicates the samples collected in this study. In the note followed by species name, for example, TPE-A-B-C, TPE indicates the city of the collection site, A indicates the random number for each collection site, B indicates the ordinal number of mosquitoes in each collection site, and C indicates the two haplotypes of individual mosquito.

**Figure 8.**
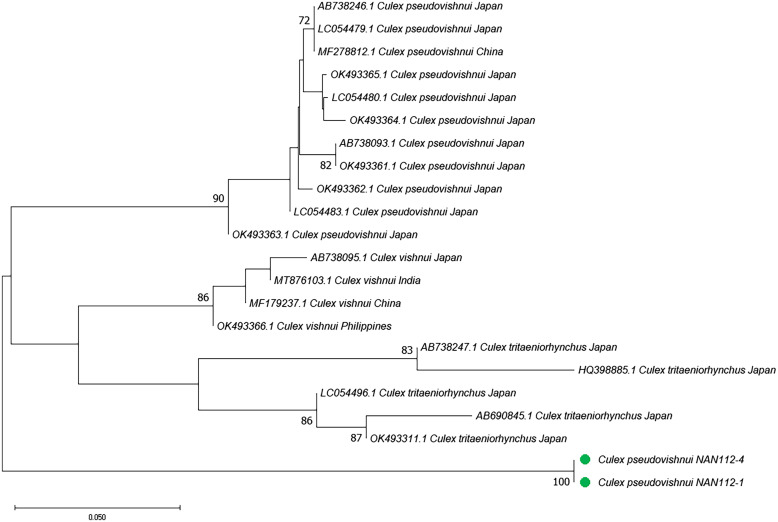
Phylogenetic analysis of *Culex pseudovishnui* based on cytochrome c oxidase subunit I (COI) sequence using neighbor-joining. Pairwise deletion, leading to 572 positions of 22 nucleotide sequences in the final dataset, were analyzed. The rate variation among sites was modeled with a γ distribution (shape parameter 0.09). The bootstrap value (1,000 replicates) above 70 is shown at nodes. The green circle indicates the samples collected in this study. In the note followed by species name, for example, TPE-A-B, TPE indicates the city of the collection site, A indicates the random number for each collection site, B indicates the ordinal number of mosquitoes in each collection site.

The *Cx. pseudovishnui* isolates from Taiwan and Japan were grouped into two clades (Figures 1 and 2). This inspired us to analyze further the relationship between the strains of this species from Taiwan and other countries. We constructed the phylogenetic trees based on the rDNA sequences of three *Cx*. *pseudovishnui* strains from Taiwan and Japan each. Phylogenetic analysis demonstrated that *Cx*. *pseudovishnui* mosquitoes from Taiwan and Japan clustered into two different clades (Figure 7 and Supplemental Figure 5). When we constructed the trees based on COI, similar results were observed. The sequence of *Cx. pseudovishnui* from Japan and China constituted a monophyletic clade; however, the *Cx. pseudovishnui* from Taiwan compressed a single clade (Figure 8 and Supplemental Figure 6). The rDNA sequences of three *Cx*. *pseudovishnui* strains collected from Nantou, Taiwan, showed less similarity (87–89%) with those of *Cx. pseudovishnui* (CP-33), *Cx. tritaeniorhynchus* (CT-22), and *Cx. vishnui* (CV-622) strains from Japan (Table 3). The COI sequences of two *Cx*. *pseudovishnui* strains collected from Nantou, Taiwan, also showed less similarity (92–94%) with those of *Cx. pseudovishnui* strains from Japan and China. The results shown in Figures 1, 2, 7, and 8 suggested a genetic gap between *Cx*. *pseudovishnui* strains from Taiwan, Japan, and China, implying that they probably belong to different species.

**Table 3:** Comparison of rDNA sequence of the strain of *Culex pseudovishnui* from Taiwan with the sequences retrieved from the NCBI database of *Culex pseudovishnui*, *Culex tritaeniorhynchus*, and *Culex vishnui* from Japan

*Culex pseudovishnui*	*Culex pseudovishnui* CP-33 (%)	*Culex tritaeniorhynchus* CT-22 (%)	*Culex vishnui* CV-622 (%)
NAN112-1-1	88.4	87.7	87.9
NAN112-1-2	88.1	87.5	87.3
NAN112-4	88.4	87.6	88

NCBI = National Center for Biotechnology Information; rDNA = ribosomal DNA and the internal transcribed spacer.

We analyzed the intra- and interspecific divergence of rDNA and COI sequences among selected *Culex* samples and reference databases. For rDNA (Table 4), all the rDNA intraspecific divergence of *Cx. annulus* strains from Taiwan and China, *Cx. vishnui* strain from Japan, *Cx. tritaeniorhynchus,* and *Cx. pseudovishnui* from Taiwan and Japan were less than 0.01863. The interspecific divergence between *Cx. annulus* and *Cx. vishnui* ranged from 0.00408 to 0.01398. However, the interspecific divergence for *Cx. pseudovishnui* from Taiwan and Japan was 0.07377, which was 3.96- and 6.03-fold higher than the variability within each strain. For COI (Table 5), all the intraspecific divergence of *Cx. annulus* strain from Taiwan and *Cx. vishnui* strains, *Cx. tritaeniorhynchus* Groups I and II, and *Cx. pseudovishnui* from Taiwan and other countries was less than 0.01220. The interspecific divergence for *Cx. annulus* from Taiwan and *Cx. vishnui* was 0.00516, which is comparable to their intraspecific divergence (0.00503 and 0.00516). The interspecific divergence for *Cx. pseudovishnui* from Taiwan and other countries was 0.07609, which was 7.06- and 44.5-fold higher than the variability within both strains. The interspecific divergence for both groups of *Cx. tritaeniorhynchus* was 0.04758, which was 3.9- and 4.5-fold higher than the variability within each group.

**Table 4:** The intra- and interspecific divergence between 155 rDNA sequences of *Culex annulus*, *Culex vishnui*, *Culex tritaeniorhynchus*, and *Culex pseudovishnui*

Species (*n*)	*Culex annulus* Taiwan	*Culex annulus* China	*Culex vishnui* Japan	*Culex tritaeniorhynchus*	*Culex pseudovishnui* Taiwan	*Culex pseudovishnui* Japan
*Culex annulus* Taiwan (76)	0.00522	–	–	–	–	–
*Culex annulus* China (2)	0.01134	0.00155	–	–	–	–
*Culex vishnui* Japan (6)	0.00408	0.01398	0.00188	–	–	–
*Culex tritaeniorhynchus* (65)	0.05771	0.05850	0.05894	0.00666	–	–
*Culex pseudovishnui* Taiwan (3)	0.07267	0.06981	0.07430	0.07791	0.01223	–
*Culex pseudovishnui* Japan (3)	0.04142	0.03950	0.04273	0.05722	0.07377	0.01863

rDNA = ribosomal DNA and the internal transcribed spacer.

**Table 5:** The intra- and interspecific divergence between 110 COI sequences of *Culex annulus*, *Culex vishnui*, *Culex tritaeniorhynchus*, and *Culex pseudovishnui*

Species (*n*)	*Culex annulus* Taiwan	*Culex vishnui*	*Culex tritaeniorhynchus* Group I	*Culex tritaeniorhynchus* Group II	*Culex pseudovishnui* Taiwan	*Culex pseudovishnui*
*Culex annulus* Taiwan (2)	0.00516	–	–	–	–	–
*Culex vishnui* (13)	0.00516	0.00503	–	–	–	–
*Culex tritaeniorhynchus* Group I (33)	0.05095	0.05171	0.01053	–	–	–
*Culex tritaeniorhynchus* Group II (49)	0.05237	0.05516	0.04758	0.01220	–	–
*Culex pseudovishnui* Taiwan (2)	0.07203	0.07437	0.07148	0.08116	0.00171	–
*Culex pseudovishnui* (11)	0.05083	0.05397	0.06805	0.06225	0.07609	0.01077

COI = cytochrome c oxidase subunit I.

## DISCUSSION

In the present study, we performed molecular analysis based on rDNA and COI to distinguish the *Cx. vishnui* subgroup in Taiwan. This investigation showed that the *Cx. annulus* strains from Taiwan clustered with the *Cx. annulus* from China and the *Cx*. *vishnui* from Japan and other Asian countries, suggesting a frequent genetic interflow among them and that they are likely to be the same species. Although rDNA sequences of *Cx*. *tritaeniorhynchus* mosquito strains from Taiwan, Japan, and China displayed a high sequence similarity and were grouped in the same clade, the result based on COI suggested that two genetically independent taxa were revealed in Asian *Cx*. *tritaeniorhynchus*. Our data show that there is a genetic gap between *Cx*. *pseudovishnui* in Taiwan, Japan, and China, implying that the Taiwanese population is probably either a unique strain or a sibling species of those in Japan and China.

In Taiwan, *Cx. annulus* is commonly distributed island-wide and is the predominant species with *Cx. tritaeniorhynchus* as a vector for JEV. Previous studies have repeatedly recognized the *Cx. vishnui* and *Cx. annulus* strains in Taiwan as different species.^4^^,^^6^^,^^36^^,^^37^ The morphological similarities shared by these two species indicate taxonomic issues. Our phylogenetic analysis, based on rDNA and COI sequences, revealed that the *Cx. vishnui* from Japan and *Cx. annulus* from Taiwan and China clustered together. A high sequence identity, including a few samples with 100% identity, was also observed among these three populations. The previous study suggested that intraspecific divergence in mosquitoes may vary from 0% to 2% (0–0.02).^38^ The interspecific divergences of rDNA between *Cx. annulus* strains from Taiwan and China and the *Cx. vishnui* strain from Japan were between 0.00408 and 0.01398, respectively. The interspecific divergence of COI between the *Cx. annulus* strain from Taiwan and the *Cx. vishnui* strains from other countries was 0.00516, which was comparable to the within-divergence of *Cx. vishnui* (0.00503) and *Cx. annulus* (0.00516) (Tables 4 and 5). These observations suggest that these strains possess high genetic similarity and agree with the well-supported group of *Cx. annulus* and *Cx. vishnui* in our phylogenetic result (98% in rDNA and 100% in COI). Our finding was also consistent with a previous study by Zhao et al.,^39^ who reported that *Cx. annulus* is a synonym of *Cx. vishnui* in China. These results indicate that the morphological identity of the *Cx. vishnui* mosquito strains and *Cx. annulus* strains from Taiwan and China are genetically similar and point out that there is frequent genetic interflow between them and that they share a gene pool. Our results are also consistent with those of a previous study that reported a high similarity (98–100%) in the ITS2 sequences and RNA secondary structures between the *Cx. vishnui* strain from Japan and the *Cx. annulus* strain from China.^40^ This observation also implies that both strains belong to the same species.

*Culex tritaeniorhynchus* has replaced *Cx. annulus* as the primary vector of JEV in Taiwan. The *Cx. tritaeniorhynchus* strain from distinct geographical areas with ecological, biological, and behavioral variations showed high sequence similarity, as demonstrated in previous studies using COI.^29^^,^^41^ In this study, the indistinguishable *Cx. tritaeniorhynchus* rDNA sequences between Taiwan, Japan, and China in a monophyletic clade differed from the tree constructed by COI, which showed two divergent lineages among *Cx*. *tritaeniorhynchus* collected from different countries. Also, the intraspecific divergence of rDNA is 0.00666, suggesting a high similarity. However, the interspecific divergence of COI of these two lineages was 0.04758, which is 3.9- and 4.5-fold higher than the intraspecific divergence of each group (Tables 4 and 5). The values do not surpass the 10-fold increase of the between-species divergence of the closest species to the within-species divergence.^42^ One explanation is that *Cx. tritaeniorhynchus* exhibited high nucleotide diversity.^43^ However, it is possible that these two lineages separated recently.^44^ The controversial results between rDNA and COI of *Cx. tritaeniorhynchus* was similar to that of previous studies, in which the COI tree topology of *Anopheles hinesorum* was distinct from those constructed by the nuclear gene targets.^29^ These observations are explainable because mitochondrial DNA was believed to exhibit a higher evaluation rate than nuclear genes.^25^ The revealed hidden taxa based on COI in morphologically identified *Cx. tritaeniorhynchus* in this study is consistent with a previous study that divided *Cx. tritaeniorhynchus* in Asia into the *Ct-*J type, which inhabits most of Japan’s territory, and the *Ct-*C type, which inhabits the Asian region (including Taiwan) except for Japan.^30^ This previous study also suggested long-distance overseas migration of *Ct*-C type *Cx. tritaeniorhynchus* from China into Japan. However, in this study, we detected two samples from Taiwan clustered with the *Ct-*J type, suggesting the possibility of bilateral migration of the *Cx. tritaeniorhynchus* strain between Taiwan and Japan. Because we detected only two samples, further surveillance should be conducted in the future. In addition, it is unclear whether these two hidden taxa are independent species, which depends on reproductive isolation, and deserves further understanding.^30^ On the other hand, our phylogenetic results and genetic divergence of *Cx. tritaeniorhynchus* based on rDNA implies that gene flow among these populations from different Asian countries was very high. We detected rDNA sequences clustered in the same clade with high sequence similarity among *Cx. tritaeniorhynchus* strains from Taiwan and Japan. The result showed that the rDNA sequence of one specimen (PTH156-3-1) is identical to that of Japan CT-22, collected in Okinawa of the Ryukyu Archipelago, the closest territory of Japan to Taiwan. Moreover, the COI sequences from the Taiwan population of *Cx. tritaeniorhynchus* shared the same pools with those from Japan and other Asian countries, which also coincides with this argument.

Passive transport, such as maritime transport and wind-mediated transmission, is a typical mode through which mosquitoes spread across ocean boundaries. Previous studies have documented the migration of *Anopheles*, *Aedes*, and *Culex* mosquitoes via craft or cargo ship transportation between continents.^15^^,^^16^^,^^45^ Long-distance travel of *Culex* mosquitoes causing Japanese encephalitis has been reported as another mode of mosquito introduction. For example, *Culex annulirostris* Skuse from New Guinea and *Cx. tritaeniorhynchus* from East Timor invaded Australia via wind-mediated migration.^29^^,^^46^ In addition, *Cx. annulirostris* mosquitoes were collected at an altitude of up to 310 m, with estimated displacements of 594–648 km,^47^ and *Cx. tritaeniorhynchus* was sampled at altitudes of 150–380 m, implying that invasions into China and India were likely associated with wind-mediated migration.^48^^,^^49^ The overseas migration of *Cx. tritaeniorhynchus* from China to Japan was supported by meteorological data.^30^ Taken together, we propose that the interactions of mosquitoes between continents frequently cause high gene flow among mosquitoes. However, the possibility of the incursion of virus-infected mosquitoes through long-distance travel, such as wind-blown travel, requires further elucidation.^30^

The *Cx. pseudovishnui* in Taiwan formed a lineage distinct from those collected in Japan, China, Philippines, and India. The identity of the rDNA genes between Taiwan and Japan was only 88% (Table 3), and the identity of the COI genes between Taiwan and other countries was 92–94%. The rDNA sequence was also different from that of *Cx. tritaeniorhynchus* and *Cx. vishnui* (Table 3). The intraspecific divergences of rDNA and COI ranged from 0.01223 to 0.01863 and from 0.00171 to 0.01077, respectively, which conformed to the general understanding that intraspecific distance in mosquitoes was less than 2%.^38^ The interspecific divergences of rDNA and COI between *Cx. pseudovishnui* strains from Taiwan and Japan were 0.07377 and 0.07609, respectively, which suggests the existence of higher sequence variations (Tables 4 and 5). Relevant research has been conducted on *Cx. pseudovishnui* strains from Taiwan, and their morphological features have been documented.^1^ A distinct form was reported as *Culex neovishnui*, with a different prothoracic hair pattern at the larval stage compared with that of *Cx. pseudovishnui*.^50^ Later, *Cx. neovishnui* Lien and *Cx. pseudovishnui* species were believed to be the same when these two species were morphologically reidentified by a Japanese scientist.^51^ These previous studies have shown the taxonomic status of *Cx. pseudovishnui* as puzzling in Taiwan. In the present study, we observed that the *Cx. pseudovishnui* strains from Taiwan, Japan, and other Asian countries were genetically different and constituted distinctive conspecific clusters, implying that the Taiwanese population was probably either a unique strain or a sibling species. However, we only obtained the adult of *Cx. pseudovishnui,* which is difficult to distinguish from *Cx. neovishnui*. Moreover, the *Cx. pseudovishnui* in Taiwan is very rare, with only three samples collected in this study. Therefore, more samples are needed to draw solid conclusions.

## CONCLUSION

In conclusion, *Cx. annulus*, *Cx. tritaeniorhynchus*, and *Cx. pseudovishnui* are morphologically similar species of the *Cx. vishnui* subgroup, usually confused with each other in field surveillance because of overlapping features, particularly *Cx. annulus* and *Cx. tritaeniorhynchus*, the vectors of JEV. Here, the molecular method based on rDNA and COI was conducted to distinguish these species. Comparisons of the rDNA and COI sequences of these species in different geographical locations suggests that *Cx. annulus* strains from Taiwan and China and *Cx. vishnui* from Japan and other Asian countries likely belong to the same species. Our results revealed the two hidden taxa based on the COI sequence of morphologically identified *Cx. tritaeniorhynchus* and suggest a frequent gene flow between the strains of *Cx. tritaeniorhynchus* from Taiwan and other Asian countries. We also identified the unique taxonomic status of *Cx. pseudovishnui* in Taiwan. Here, we added valuable information to the molecular taxonomy of the *Cx. vishnui* subgroup in Taiwan, which is necessary for vector control operations and tracing evolutionary processes.

## Supplemental Materials

10.4269/ajtmh.23-0285 Supplemental Tables

10.4269/ajtmh.23-0285 Supplemental Figures
