# Supplementary material for: Molecular Identification of Species Belonging to Culex vishnui Subgroup (Diptera: Culicidae), Vectors of Japanese Encephalitis Virus, in Taiwan
Source: Am J Trop Med Hyg. 2024 Sep 10;111(5):988–99. doi: 10.4269/ajtmh.23-0285 (PMC11542521; doi:10.4269/ajtmh.23-0285)
Supplement: Supplemental Tables [file tpmd230285.SD1.pdf]

Table S1. GenBank Assession number of rDNA and COI sequencies from NCBI database

Species	rDNA	COI	Country
Cx. annulus	AF453488		China
	KF499144		China
Cx. tritaneorhynchus	U33041		Taiwan
	U33042		Taiwan
	AF165896		Japan
	AF165897		Japan
	EF545175		China
	EF545158		China
		LC054496	Japan
		OK493311	Japan
		OK493312	Japan
		OK493313	Japan
		OK493314	Japan
		OK493315	Japan
		OK493316	Japan
		OK493317	Japan
		OK493318	Japan
		AB690848	Japan
		AB690849	Japan
		AB690850	Japan
		LC054497	Japan
		AB690847	Japan
		LC054499	Japan
		OK493319	Japan
		AB690855	Japan
		OK493320	Japan
		AB738247	Japan
		LC054498	Japan
		AB690854	Japan
		LC054501	Japan
		AB690845	Japan
		AB690853	Japan
		OK493321	Japan
		OK493322	Japan
		OK493323	Japan
		OK493324	Japan
		AB690846	Japan
		AB690857	Japan
		OK493325	Japan
		OK493326	Japan
		OK493327	Japan
		OK493328	Japan
		OK493329	Japan
		OK493330	Japan
		OK493331	Japan

		OK493332	Japan
		OK493333	Japan
		OK493334	Japan
		OK493335	Japan
		OK493336	Japan
		OK493337	Japan
		OK493338	Japan
		OK493339	Japan
		OK493340	Japan
		OK493341	Japan
		AB690852	Japan
		LC054500	Japan
		OK493342	South Korea
		OK493343	South Korea
		OK493344	China
		OK493345	China
		OK493346	Taiwan
		OK493347	Taiwan
		OK493348	Taiwan
		OK493349	Philippines
		HQ398885	Thailand
		OK493350	Vietnam
		OK493351	Vietnam
		OK493352	Vietnam
		OK493353	Vietnam
		OK493354	Vietnam
		OK493355	Vietnam
		OK493356	Vietnam
		OK493357	Vietnam
		OK493358	Vietnam
		OK493359	Vietnam
		OK493360	Vietnam
	AF165894		Japan
	AF165895		Japan
	AF453495		China
	AF453496		China
	AF453497		China
	AF453498		China
	KF499144		China
		LC054480	Japan
Cx. psuedovishnui		LC054479	Japan
		OK493361	Japan
		OK493362	Japan
		OK493363	Japan
		AB738246	Japan
		LC054483	Japan
		OK493364	Japan
		OK493365	Japan

		AB738093	Japan
	AF165898		Japan
	AF165899		Japan
	AF165900		Japan
		MT876103	India
		AB738195	Japan
		AB738095	Japan
		AB738296	Japan
		AB738149	Japan
Cx. vishnui		MF179237	China
		MF179238	China
		MF179239	China
		MF179240	China
		OK493366	Philippines
		OK493367	Philippines
		OK493368	Philippines
		OK493369	Philippines
		OK493370	Philippines
