# Supplementary material for: Molecular Identification of Species Belonging to Culex vishnui Subgroup (Diptera: Culicidae), Vectors of Japanese Encephalitis Virus, in Taiwan
Source: Am J Trop Med Hyg. 2024 Sep 10;111(5):988–99. doi: 10.4269/ajtmh.23-0285 (PMC11542521; doi:10.4269/ajtmh.23-0285)
Supplement: Supplemental Figures [file tpmd230285.SD2.pdf]

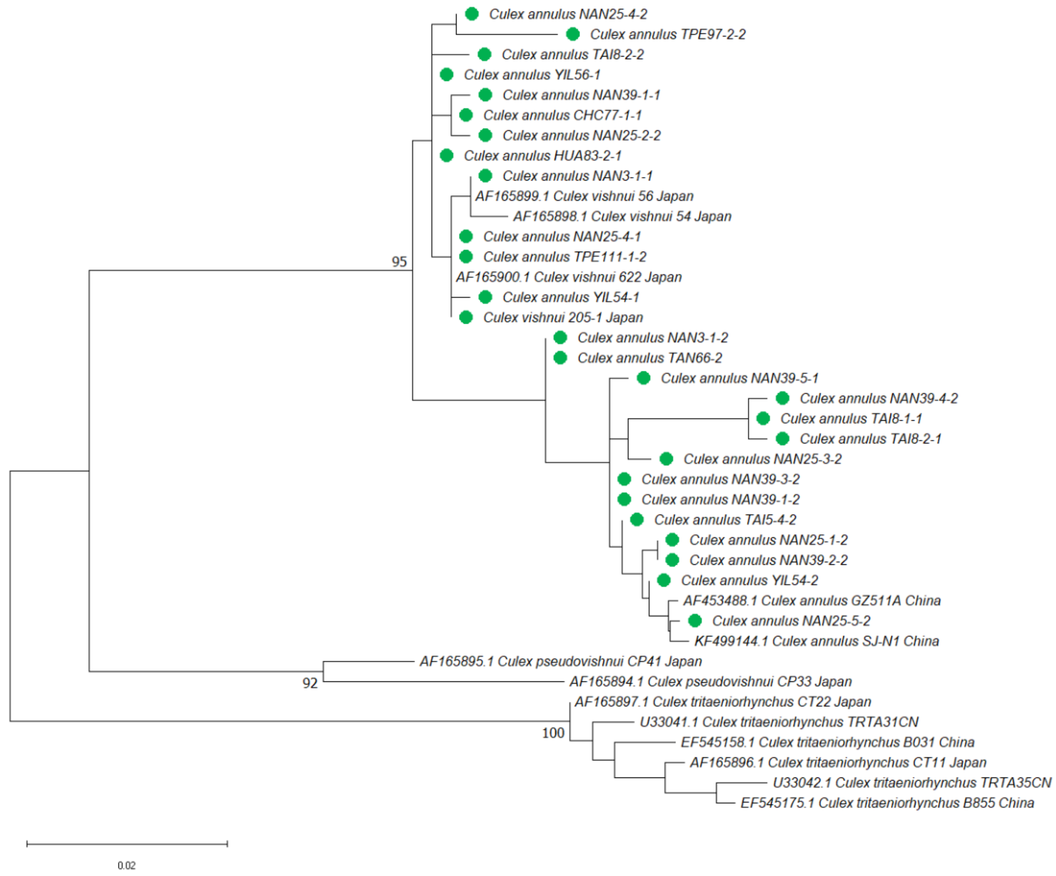

Figure S1. Phylogenetic analysis of *Culex vishnui and Culex annulus* based on rDNA sequences using Maximum Likelihood. A total of 679 positions of 40 nucleotide sequences in the final dataset were analyzed. The bootstrap value (1000 replicates) higher than 70 is shown at nodes. Green circles indicate the samples collected in this study. Annotation after species name, for example, TPE-A-B-C, where TPE indicates the city of collection site, A indicates the random number for each collection site, B indicates the ordinal number of mosquitoes in each collection site, C indicates the 2 haplotypes of individual mosquito.

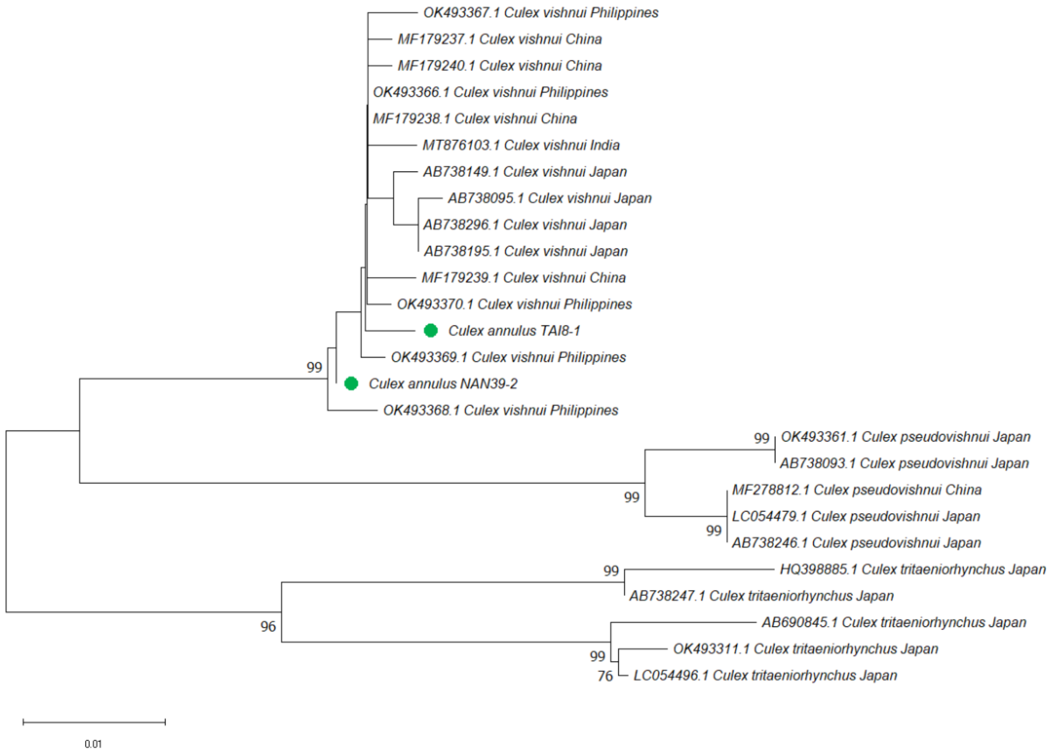

Figure S2. Phylogenetic analysis of *Culex vishnui and Culex annulus* based on COI sequences using Maximum Likelihood. A total of 618 positions of 26 nucleotide sequences in the final dataset were analyzed. The bootstrap value (1000 replicates) higher than 70 is shown at nodes. The green circle indicates the samples collected in this study. Annotation after species name, for example, TPE-A-B, where TPE indicates the city of collection site, A indicates the random number for each collection site, B indicates ordinal number of mosquitoes in each collection site.

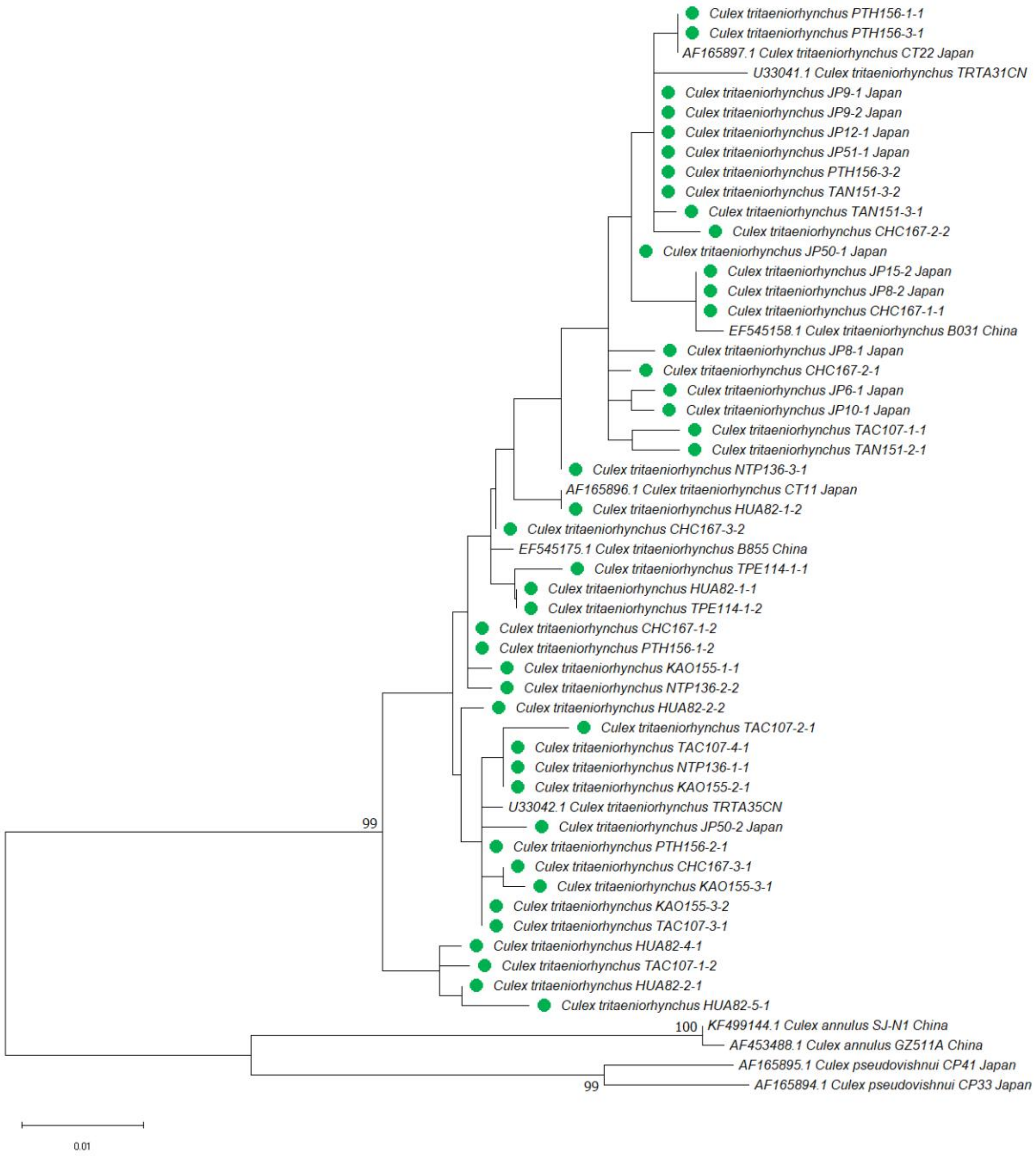

Figure S3. Phylogenetic analysis of *Culex tritaeniorhynchus* based on rDNA sequences using Maximum Likelihood. A total of 696 positions of 55 nucleotide sequences in the final dataset were analyzed. The bootstrap value (1000 replicates) above 70 is shown at nodes. The green circle indicates the samples collected in this study. The note followed by species name, for example, TPE-A-B-C, where TPE indicates the city of collection site, A indicates the random number for each collection site, B indicates the ordinal number of mosquitoes in each collection site, and C indicates the 2 haplotypes of individual mosquito.

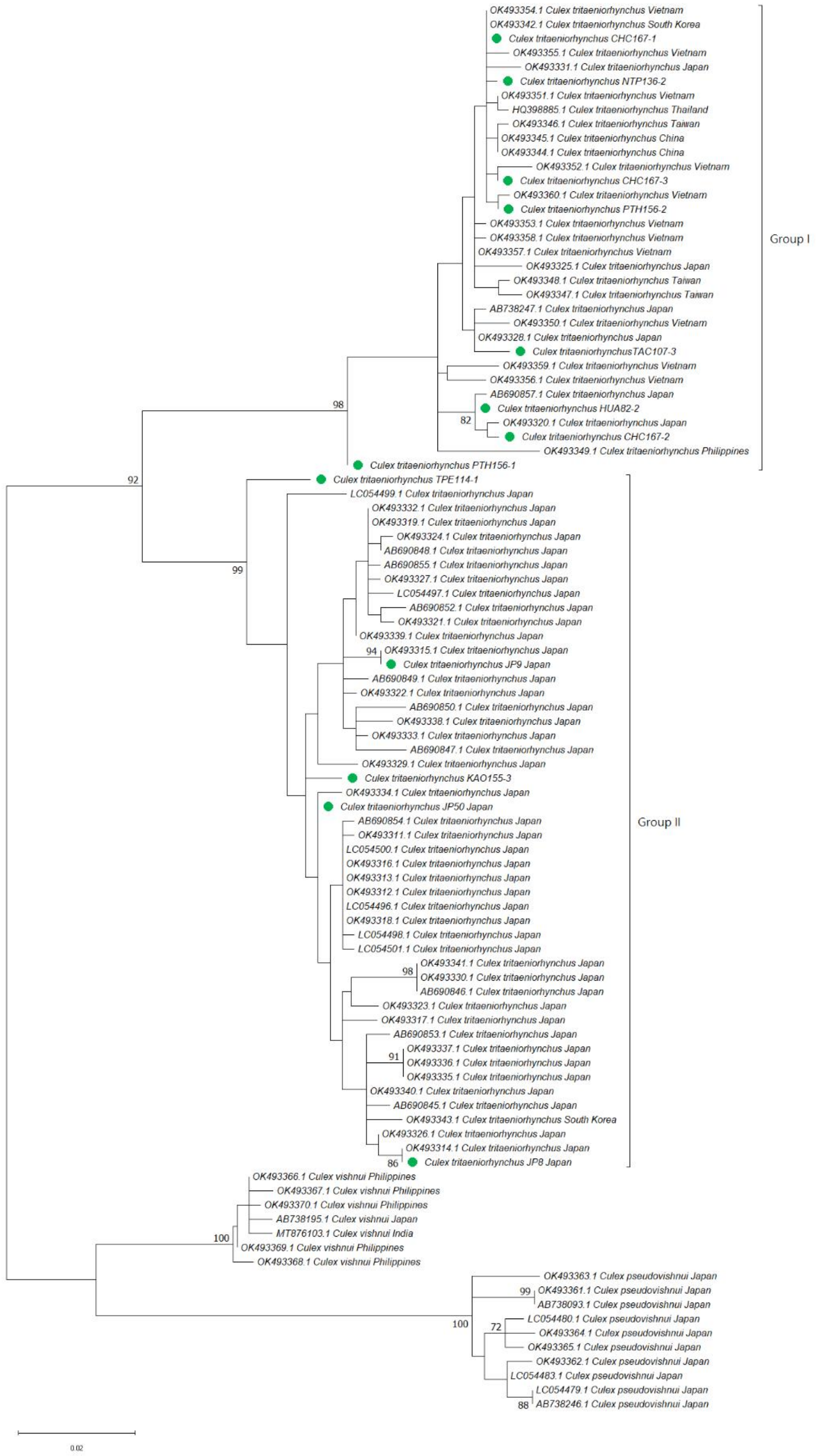

Figure S4. Phylogenetic analysis of *Culex tritaeniorhynchus* based on COI sequences using Maximum Likelihood. A total of 587 positions of 99 nucleotide sequences in the final dataset were analyzed. The bootstrap value (1000 replicates)

higher than 70 is shown at nodes. The green circle indicates the samples collected in this study. Annotation after species name, for example, TPE-A-B, where TPE indicates the city of collection site, A indicates the random number for each collection site, B indicates ordinal number of mosquitoes in each collection site.

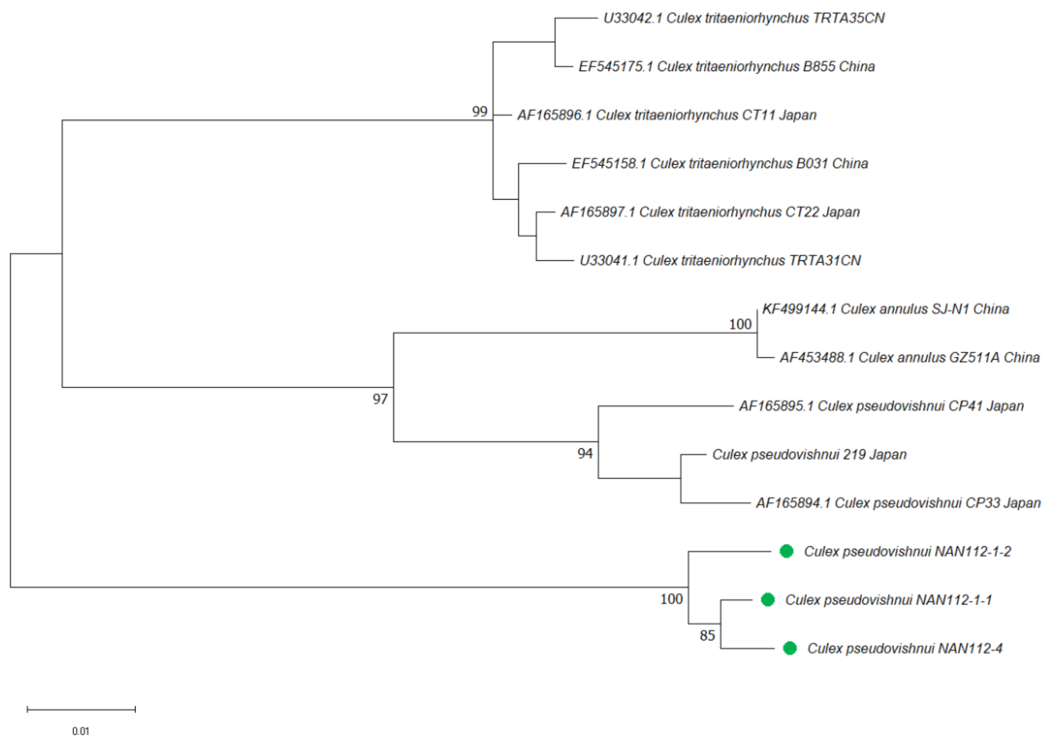

Figure S5. Phylogenetic analysis of *Culex pseudovishnui* based on rDNA sequence using Maximum Likelihood. A total of 692 positions of 14 nucleotide sequences in the final dataset were analyzed. The bootstrap value (1000 replicates) above 70 is shown at nodes. The green circle indicates the samples collected in this study. The note followed by species name, for example, TPE-A-B-C, where TPE indicates the city of the collection site, A indicates the random number for each collection site, B indicates the ordinal number of mosquitoes in each collection site.

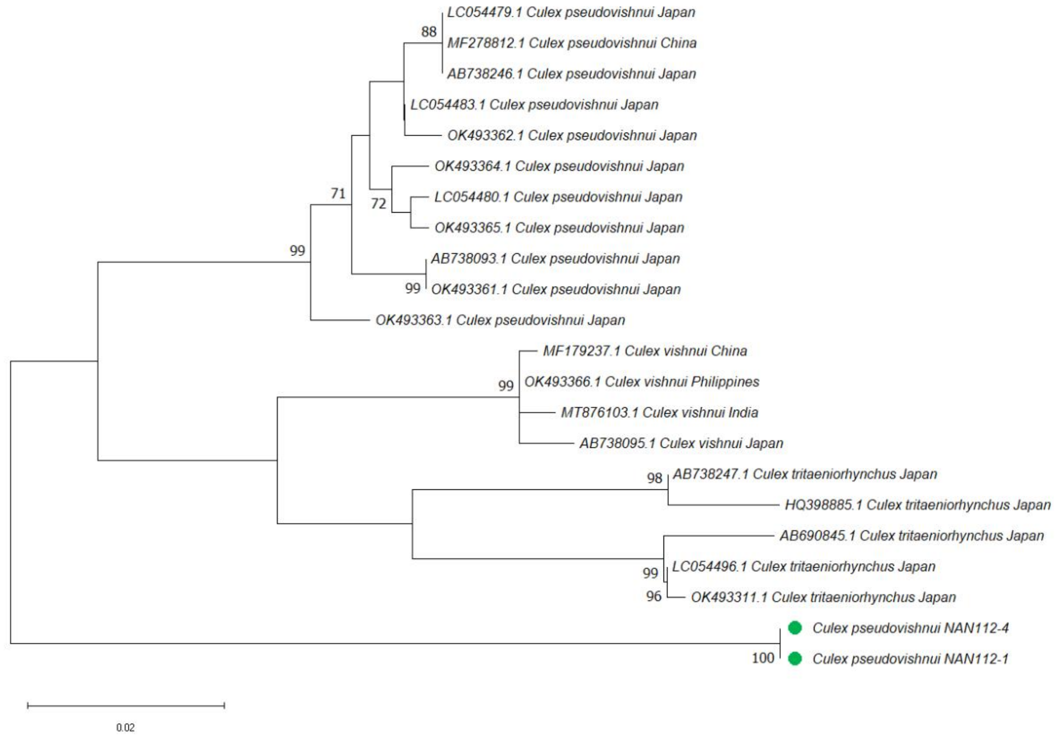

Figure S6. Phylogenetic analysis of *Culex pseudovishnui* based on COI sequence using Maximum Likelihood. A total of 572 positions of 22 nucleotide sequences in the final dataset were analyzed. The bootstrap value (1000 replicates) above 70 is shown at nodes. The green circle indicates the samples collected in this study. The note followed by species name, for example, TPE-A-B-C, where TPE indicates the city of the collection site, A indicates the random number for each collection site, B indicates the ordinal number of mosquitoes in each collection site.
